# The impact of self-directed learning experience and course experience on learning satisfaction of university students in blended learning environments: the mediating role of deep and surface learning approach

**DOI:** 10.3389/fpsyg.2023.1278827

**Published:** 2024-01-08

**Authors:** Meng Hua, Lin Wang, Jun Li

**Affiliations:** ^1^International office, Xuzhou Kindergarten Teachers College, Xuzhou, Jiangsu, China; ^2^Graduate School, Lyceum of the Philippines University, Trias, Cavite, Philippines; ^3^School of Preschool and Special Education, Xuzhou Kindergarten Teachers College, Xuzhou, Jiangsu, China; ^4^School of International Studies, NingboTech University, Ningbo, Zhejiang, China

**Keywords:** self-directed learning experience, course experience, deep learning approach, surface learning approach, learning satisfaction, blended learning, university students

## Abstract

**Introduction:**

With the rapid development of technology and the evolution of educational ideas, the blended learning model has become the new norm in higher education. Therefore, based on Biggs’ learning process theory, this study aims to investigate the relationships between learning experience, learning approaches, and learning satisfaction of university students within the Chinese blended learning context to explore the dynamic process and mechanism of blended learning.

**Methods:**

The Chinese modified versions of the Self-Rating Scale of Self-Directed Learning, the Course Experience Questionnaire, and the Revised Study Process Questionnaire were administered to 939 Chinese university first-grade students (444 men, 495 women). The data were analyzed by using the covariance-based structural equation modeling (CB-SEM) technique.

**Results:**

The results demonstrated that, among Chinese university students, there were significant correlations between the self-directed learning experience, the course experience, the deep learning approach, the surface learning approach, and learning satisfaction. Additionally, the learning approaches mediated the association between the self-directed learning experience and learning satisfaction and between the course experience and learning satisfaction.

**Conclusion:**

This study provides insight into the facilitative effect of university students’ self-directed learning experience and course experience on their learning satisfaction and how this effect is triggered through the mediating role of different learning approaches with the blended learning context. This study shows the learning behaviors and psychology in a blended learning environment, thus revealing the new learning characteristics of university students by integrating the self-learning characteristics of blended learning into the framework of learning process theory. The findings contribute to assisting blended learning providers in delivering targeted interventions to enhance students’ learning satisfaction.

## 1 Introduction

With the rapid development of technology and the evolution of educational ideas, blended learning, as an innovative learning mode, has gradually emerged and been widely adopted in education (Köse, 2010; Kaplan and Haenlein, 2016). Developed by E-learning, blended learning combines face-to-face and online learning, in which 30–70% of the learning content is online (Bonk and Graham, 2012). This approach effectively integrates teacher guidance, student autonomy, and the utilization of technological tools, providing students with a more personalized, flexible, and diversified learning experience. It promotes interaction, collaboration, and development of critical thinking skills. Blended learning in the initial development stage was mainly understood as a new way of learning, emphasizing the core role of technology in learning (Miyazoe and Anderson, 2010; Feng et al., 2018). As blended teaching has become a traditional means of teaching reform, researchers changed the concentration to how to use this model to promote learning practice. From this perspective, many scholars have paid attention to teachers’ professional competence, instructional preparation, teaching design, implementation, and evaluation (Akyol et al., 2009; Donnelly, 2010; Korr et al., 2012; Garrison and Vaughan, 2013; Keengwe and Kang, 2013; Xiao, 2016).

With the change of research focus, scholars have begun to pay more attention to the changes brought to students and the support for students learning by blended learning from the perspective of learners (Smith, 2015). Firstly, some scholars have explored students’ personal learning experience under a blended learning environment (Cheon et al., 2012; Osgerby, 2013; Llorente et al., 2016). In addition, some researchers have studied the effect of blended learning. They have found that blended learning can effectively improve students’ academic performance and enhance their learning motivation, self-efficacy, critical thinking ability, and learning satisfaction (López-Pérez et al., 2011; Garrison and Vaughan, 2013; Sáiz-Manzanares et al., 2020). Furthermore, the approaches and strategies in blended learning have also been explored. Some argued that the blended model promoted deep learning so that learners were better able to engage in blended learning activities (Halverson and Graham, 2019), while another view was that a blended curriculum made it harder for students to participate, less motivated to learn, and superficially engaged because switching between the traditional and online models had taken them too much effort (Banerjee, 2011; Shen et al., 2011). Such complex and abundant research achievement about blended learning practice would seem fruitful grounds for researchers who study from the perspective of learners.

However, the above research from learners’ perspective on the three aspects of blended learning was static and fragmented, and the dynamic mechanism of blended learning had yet to receive sustained attention in the blended learning literature (Zhao et al., 2019; Liu et al., 2021; Ma et al., 2021; Zhang et al., 2022). This was also one of the critical reasons for the contradiction of research results. Biggs’ learning process model is a comprehensive framework that can combine these different aspects of blended learning from the perspective of learners (Biggs, 1989). Because it and blended teaching both align with the constructivism learning theory, the model is highly suitable for examining blended learning contexts (Biggs, 1989; Biggs, 1993; Donnelly, 2010; Yang and Kuo, 2021). Therefore, given that the blended model has become the new norm in higher education teaching since the end of the COVID-19 pandemic (Mahaye, 2020; Mali and Lim, 2021), the present study work with these understandings to explore the dynamic mechanism of blended learning through drawing on the accounts of the first-year university students. Specifically, this paper aims twofold: (1) to investigate the relationship between learning experience and the learning satisfaction in blended learning; (2) to seek the mediating effect of different learning approaches on learning experience and learning satisfaction in blended learning. Through such focus on the different aspects of learning, the paper advances scholarship within the emerging field of blended learning, extending recent moves to recognize the new learning characteristics of university students under the blended learning environment. In addition, by achieving these aims, this paper can serve as an anchoring point to provide better-differentiated teaching and learning support for university students taking blended courses at the policy and practical levels.

In what follows, the paper introduce Biggs’ learning process model and its applicability to blended learning contexts. Then, it review the previous literature on university students’ learning experience, learning approaches, and learning satisfaction in detail and put forward the hypotheses. The remainder of the paper then outlines the research design and findings from the study, exploring the direct effect of learning experience on learning satisfaction and the indirect effect of learning experience on learning satisfaction through different learning approaches. Lastly, the article discuss empirical and theoretical contributions, limitations, and future directions.

### 1.1 Biggs’ learning process theory and the blended learning model

**Figure 1:**
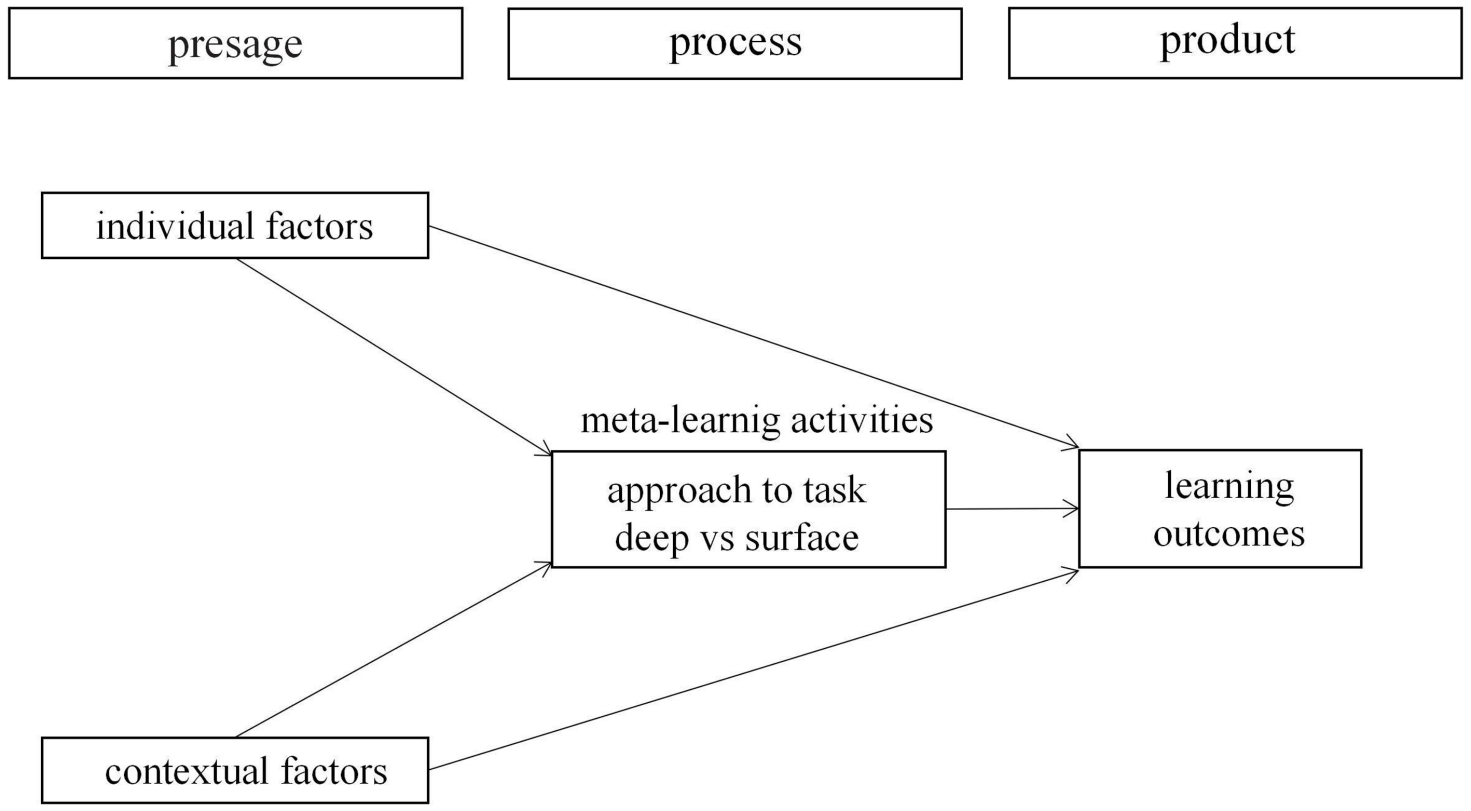
Biggs’ learning process theory.

According to Biggs’ learning process theory, the three aspects of the blended learning from the perspective of learners mentioned above belong to different constructs of learning (Biggs, 1993). However, few have combined these aspects for research (Zhao et al., 2019; Liu et al., 2021; Ma et al., 2021; Zhang et al., 2022). This is not conducive to a deep analysis of the entire dynamic system nor to accurately grasping the mechanism of blended learning of university students. Biggs’ learning process model is a comprehensive framework for analyzing the dynamic system of university students’ learning from the perspective of learners, which has been widely used in exploring the mechanisms of university students from diverse social and cultural backgrounds and different majors (Mladenovic, 2000; Reid et al., 2013; Guo et al., 2017; Ganotice and Chan, 2019; Kanashiro et al., 2020). The theory holds that there are three constructs of learning, which are presage, process, and product. Presage factors not only include individual factors such as students’ particular abilities, experience, values, expectations, motivations, and demographics but also refer to contextual factors such as course structure, teaching skills, and methods of teaching and assessment, all of which generate a “climate” for learning (Biggs, 1989; Kanashiro et al., 2020). The product is learning outcomes which can be measured by objective indicators or by subjective perception. Students interpret the learning context in the light of their perception, giving rise to a meta-cognitive activity focusing on learning itself, not on the contents of learning. This activity of “meta-learning,” which refers to the learning process, enables students to derive their approaches to learning, and learning outcomes are determined by approaches adopted (Biggs, 1989). In short, presage factors decide how students approach a particular task, which in turn mediates or affects outcomes achieved. Presage factors can also directly decide learning outcomes. Figure 1 provides a description of Biggs’ learning process theory in higher education.

This theory is also applicable to research on blended learning contexts. Firstly, it posits that learners in blended learning contexts are autonomous agents who proactively choose information from their perceived environment and build new knowledge system on the basis of their existing constructivist perspective and prior knowledge (Biggs, 1993). This is consistent with the essence of blended learning, which emphasizes that learners develop their understanding and knowledge by actively engaging in actions and reflecting upon them in interactive classroom settings (Donnelly, 2010). Secondly, the roles of students and instructors are congruent in both Biggs’ learning process theory and the blended learning model. They both stress the harmonious integration of teacher-led and student-centered roles. They not only emphasize the significance of individual learner’s quality and learning styles, including their autonomy, motivation, and self-efficacy (Ho et al., 2016; Jiang et al., 2021), but also attach importance to the teachers’ role as impostors, guides, facilitators, and supervisors during the instructional process (Biggs, 1989). Thirdly, both advocate for the significance of instructional contexts. In Biggs’ learning process model, diversified and nurturing learning contexts encompass all teacher-controlled factors, which produce significant motivational consequences to the learning process and product (Biggs, 1989). Meanwhile, implementing blended learning provides students with immersive, supportive, constructive, and markedly participatory instructional contexts (Biggs, 1989; Yang and Kuo, 2021). Therefore, Biggs’ learning process theory provides a reliable theoretical basis which can be adapted for exploring the mechanism of blended learning in the present study.

In light of this, the present study adopts a learner-centered perspective and utilizes the learning process theory, which aligns with constructivist principles, to construct an analytical framework for university students’ blended learning. Firstly, in a blended learning environment, students generate different learning experience. One is the self-directed learning experience for online learning, and the other is the course experience for face-to-face learning. Self-directed learning is chosen for this study for two reasons: (1) it is to be a control over external learning environment, and the presage factors of Biggs’ learning process theory are also referred to students’ perception of learning environment (Guo et al., 2017; Linkous, 2021); (2) Blended learning activities for courses in Chinese universities generally start with online learning, in which students handle online learning resources, conduct online training and discussion (Zhao, 2022). When learning online, students need to mobilize their self-efficacy, self-discipline and motivation, and to plan, implement, and evaluate personal learning, which is exactly the emphasis of the self-directed learning (Linkous, 2021). Secondly, students use different learning approaches in their meta-cognitive activity focusing on learning itself. Thirdly, one of the learning outcomes is the learning satisfaction, which is one of the critical psychological characteristic in blended learning that represents students’ subjective feeling at the product level. This study conceptualizes learning satisfaction as an outcome resulting from combining students’ individual factors and contextual factors related to blended learning. Finaly, according to Biggs’ learning process theory, the particular blended learning experience leads to different on-task learning approaches, which in turn leads to different perception of learning satisfaction. Next, before proceeding with the data analysis, we will review the empirical literature on the relationships between learning experience, learning approaches, and learning satisfaction.

### 1.2 Overview of literature review and the hypotheses development

#### 1.2.1 University students’ learning satisfaction of blended learning

Blended learning is a student-centered model that attaches great importance to students’ subjective feelings. Learning satisfaction is learners’ pleasant feelings or attitudes toward learning activities (Long, 1985). As an emotional or attitudinal outcome of learning, learning satisfaction emphasizes the degree to which students’ expectations are met and the extent to which the teaching-learning process responds to students’ needs. It can predict the persistence of students’ learning and has been repeatedly regarded as a critical element reflecting the quality of subjective experience (de la Fuente et al., 2020). Several studies have indicated that the blended model can enhance students’ satisfaction and academic achievements to a certain degree in contrast to conventional face-to-face and entirely online instruction (López-Pérez et al., 2011; Henrie et al., 2015). Nevertheless, another research has demonstrated that students evaluated blended learning less favorably than the traditional model (Utts et al., 2017). This may result from the interaction between presage and process factors of blended learning (Guo and Ji, 2019; Hua and Wang, 2023). So it is necessary to analyze various factors that affect learning satisfaction of blended learning and then understand the blended learning mechanism of university students.

The notion of learning satisfaction is derived from the contemporary theory of customer satisfaction, which posits that customers are no longer mere recipients of services but rather active participants in the service system. Providing high-quality service is a shared responsibility between the service provider and the customer (Hill, 1995). In the context of the learner experience, high satisfaction with learning is also contingent upon the active participation of students and the delivery of high-quality learning by educators. Blended learning is a model that integrates traditional classroom learning with technology-mediated individual learning, thereby facilitating teaching and learning in both physical and virtual settings (Collis and Moonen, 2012). In this context, the self-directed learning experience in online environment and the course experience delivery by teachers in offline educational milieu is equally significant, and both can influence learning satisfaction (Diep et al., 2017).

#### 1.2.2 The relationship between university students’ learning experience and learning satisfaction

In blended learning, students have greater flexibility in deciding when, how, and what content and activities to engage in Milligan and Littlejohn (2014). Self-directed learning is a process by which learners guide themselves to acquire knowledge and develop the problem-solving skills necessary for learning (Geng et al., 2019). The core of the self-directed learning experience is the learners’ sense of responsibility or control over their learning (Benson, 2013). The process of self-directed learning can facilitate collaborative learning using internet communication technologies (Lee et al., 2014). Previous research has shown that the experience of technology and online tools in self-directed learning was a key individual factor in learning satisfaction (Kintu et al., 2017), and the self-efficacy experience in online self-directed learning directly affected learning satisfaction (Shen et al., 2013). The self-directed strategy in learning can significantly predict the blended cooperative learning satisfaction, and the action path can be summarized as “goal-driven, resource-promoting, evaluation-guaranteeing, and strategy-first” (Zhang, 2017, p. 564). A large-scale survey in China has found that insufficient self-control ability and low levels of information literacy may be important influencing factors leading to low learning satisfaction (Wan et al., 2020).

Another presage factor, students’ course experience, represents their perception of the external teaching environment, such as teaching quality, evaluation, workload, and teaching interaction (Diseth et al., 2006; Diseth, 2007). A previous study has identified that good teaching experience could positively predict course satisfaction in traditional classroom (Guo et al., 2017). Some scholars have also found that perceived teacher attitude, course flexibility, course quality, course usefulness, course usability, and perceived multiple evaluations were the main factors affecting their learning satisfaction in online classrooms (Arbaugh, 2000; Arbaugh and Duray, 2002; Sun et al., 2008). Many researchers have also focused on the relationship between course experience and learning satisfaction in blended learning context. Zhao and Yuan (2010) have indicated that the experience of course applicability, flexibility, and richness could influence learning satisfaction. Another study by So and Brush (2008) has shown that clear teaching guidance, teaching activities, face-to-face support, and collaborative ability were essential factors that affected students’ satisfaction with blended teaching. Blended courses provided even more opportunities for students to communicate and interact during the pandemic, thereby increasing their cognitive-affective experience with the course (Bouilheres et al., 2020; Asghar et al., 2021). This experience encouraged discussion and critical thinking, translating into increased learning satisfaction (Al Awamleh, 2020; Batista-Toledo and Gavilan, 2023). It appears that regardless of the teaching environment, there is an inextricable connection between course experience and learning satisfaction. The model of building satisfaction in the blended learning systems showed that self-efficacy and achievement goals related to the self-directed learning experience and teacher support related to the course experience were both key factors affecting students’ satisfaction (Diep et al., 2017). The fact also verifies from the side that different presage factors have a close relationship and can jointly influence learning satisfaction (Guo and Ji, 2019; Hua and Wang, 2023). Based on the literature, the following hypotheses are brought forward.

*H1*: University students’ self-directed learning experience can directly predict learning satisfaction within Chinese blended learning context.

*H2*: University students’ course experience can directly predict learning satisfaction within Chinese blended learning context.

#### 1.2.3 The relationship between university students’ learning experience and learning approaches

Students use different approaches to cope with learning tasks during learning process. The surface learning approach is directed to a concrete task and is motivated by extrinsic motivation (Biggs, 1989). This learning approach is superficial, meaning it primarily involves rote memorization with the narrow target of passing exams and obtaining academic certification (Biggs et al., 2001). The other is the deep learning approach, inspired by an internal interest in learning, characterized by gaining the most profound meaning from the learning experience, involving active learning process that relate ideas, look for patterns and principles, and obtain a deeper understanding of key concepts (Biggs, 1993). In general, the “deep” learning approach is described as actively striving to improve understanding by applying and comparing ideas, while the “surface” one involves a reproductive strategy that incorporates little attempt to integrate information. The Biggs’ learning process theory has identified that presage factors determine which approach to learning students will adopt, thereby determining the quality of learning outcomes (Biggs, 1993). Therefore, exploring the relationship between university students’ learning experience, learning approaches, and learning satisfaction with blended learning is necessary.

Previous studies have shown that students’ self-directed learning was closely related to the deep learning approach. In a specific deep learning context like problem-based learning (PBL), students were responsible for their learning, so they engaged in self-directed learning and then applied their new knowledge to the problem and reflected on what they learned and the effectiveness of the strategies employed (Scardamalia et al., 1989; Hmelo-Silver, 2004). Highly self-directed learners typically engaged more in various learning activities, including thoroughly reading online learning materials, diligently completing assigned classroom tasks, and actively planning and assessing their progress toward personal learning goals (Law et al., 2010). Therefore, individuals with good self-directed learning experience are likelier to adopt a deep learning approach. In contrast, students with poor self-directed learning experience tend to adopt a surface learning approach.

Course experience is another presage factor closely related to learning approaches, too. The positive experience of teachers’ scaffolding role in a course can encourage students’ deep thinking and promote the formation of a cognitive apprenticeship (Hmelo-Silver, 2006; Collins et al., 2018). Students’ good experience of teaching presence in a course can construct learning content, encourage student participation, promote discourse, and guide students toward a deep approach to learning (Garrison et al., 2001). However, the course experience that lacked interaction between teachers and students can lead students to adopt a surface approach in a college classroom (Guo et al., 2017). Based on the literature, the following hypotheses are put forward.

*H3*: University students’ self-directed learning experience can positively predict the deep learning approach (H3a) and negatively predict the surface learning approach (H3b) within Chinese blended learning context.

*H4*: University students’ course experience can positively predict the deep learning approach (H4a) and negatively predict the surface learning approach (H4b) within Chinese blended learning context.

#### 1.2.4 The relationship between learning approaches and learning satisfaction

The learning approaches adopted by students are closely related to learning satisfaction. Abraham (2006) asserted that “…students who adopt a deep approach to learning will be more satisfied with the course than those who adopt a surface approach” (p. 10). Bobe and Cooper (2020) have further indicated that a surface approach that involved setting narrow goals, rote memorization, lack of appropriate motivation strategies, and learning management negatively predicted students’ learning satisfaction. More research conducted in different cultural contexts with students in different disciplines and at different levels has found that a deep learning approach based on intrinsic interest in gaining knowledge and maximizing meaning positively predicted students’ learning satisfaction (Lucas, 2001; Gurpinar et al., 2013; Bobe and Cooper, 2019; de la Fuente et al., 2020; Tadesse et al., 2022). Moreover, compared to a traditional learning environment, students who used PBL as a deep learning approach in a blended learning environment had higher satisfaction ratings (Woltering et al., 2009). Based on the literature, the following hypotheses are formulated.

*H5*: University students’ deep learning approach can positively predict the learning satisfaction within Chinese blended learning context.

*H6*: University students’ surface learning approach can negatively predict the learning satisfaction within Chinese blended learning context.

#### 1.2.5 The role of learning approaches in learning experience and learning satisfaction

In recent years, an increasing number of scholars have begun to realize the bridging role of learning approaches between course experience and learning outcomes. Some confirmed that students’ experience of effective teaching (both in teaching and presence) in different teaching models was positively correlated with deep learning approaches, and the experience was significantly positively correlated with students’ overall satisfaction (Kim and Lee, 2019; Bobe and Cooper, 2020). The research conducted by Guo et al. indicated that students’ experience of learning environment directly impacted learning outcomes, and the deep learning approach indirectly mediated this impact (Guo et al., 2017). Multiple studies found that the surface learning approach mediated the relationship between course experience and learning outcomes (Diseth, 2007; Diseth et al., 2010; Trigwell et al., 2013). Although these studies have explored the relationship between course experience, learning approaches, and learning outcomes, they have only focused on the relationship between a single teaching or environmental dimension in course experience and a single learning approach. Moreover, the concentration on learning outcomes is more on the objectification of achievement rather than the subjective perception of students. This is not conducive to a comprehensive understanding of the mechanism by which course experience through different learning approaches affect learning satisfaction and may lead to inconsistent research conclusions. As Yin et al. (2014) and Yin et al. (2016) reported, a contrasting viewpoint to the above research suggested that students’ good course experience can only predict the surface learning approach rather than the deep learning approach or learning satisfaction. In addition, while studying the impact of course experience on university students’ learning approaches and outcomes, individual factors may also contribute simultaneously. According to the Biggs’ learning process model, students’ learning approaches are determined by both individual factors and contextual factors (Biggs, 1993). The subjective nature of these factors easily affect learning approaches (Vermunt and Donche, 2017). Deeper learning approaches, higher satisfaction, and higher achievement will accompany greater joint regulation of internal and external factors. In contrast, lower levels of joint regulation will determine more surface learning, lower satisfaction, and lower achievement (de la Fuente et al., 2020). Students take control of their learning self-directedly using modern information technology in the blended learning environment, so the self-directed learning experience is one of students’ typical individual factors, which can produce a joint effect with course experience. Moreover, it has already been discussed that the self-directed learning experience may be closely related to various learning approaches and learning satisfaction. In summary, the following hypotheses are proposed.

*H7*: The effect of self-directed learning experience on learning satisfaction is mediated by the deep learning approach (H7a) and the surface learning approach (H7b) in Chinese university students’ blended learning.

*H8*: The effect of course experience on learning satisfaction is mediated by the deep learning approach (H8a) and the surface learning approach (H8b) in Chinese university students’ blended learning.

### 1.3 This study

**Figure 2:**
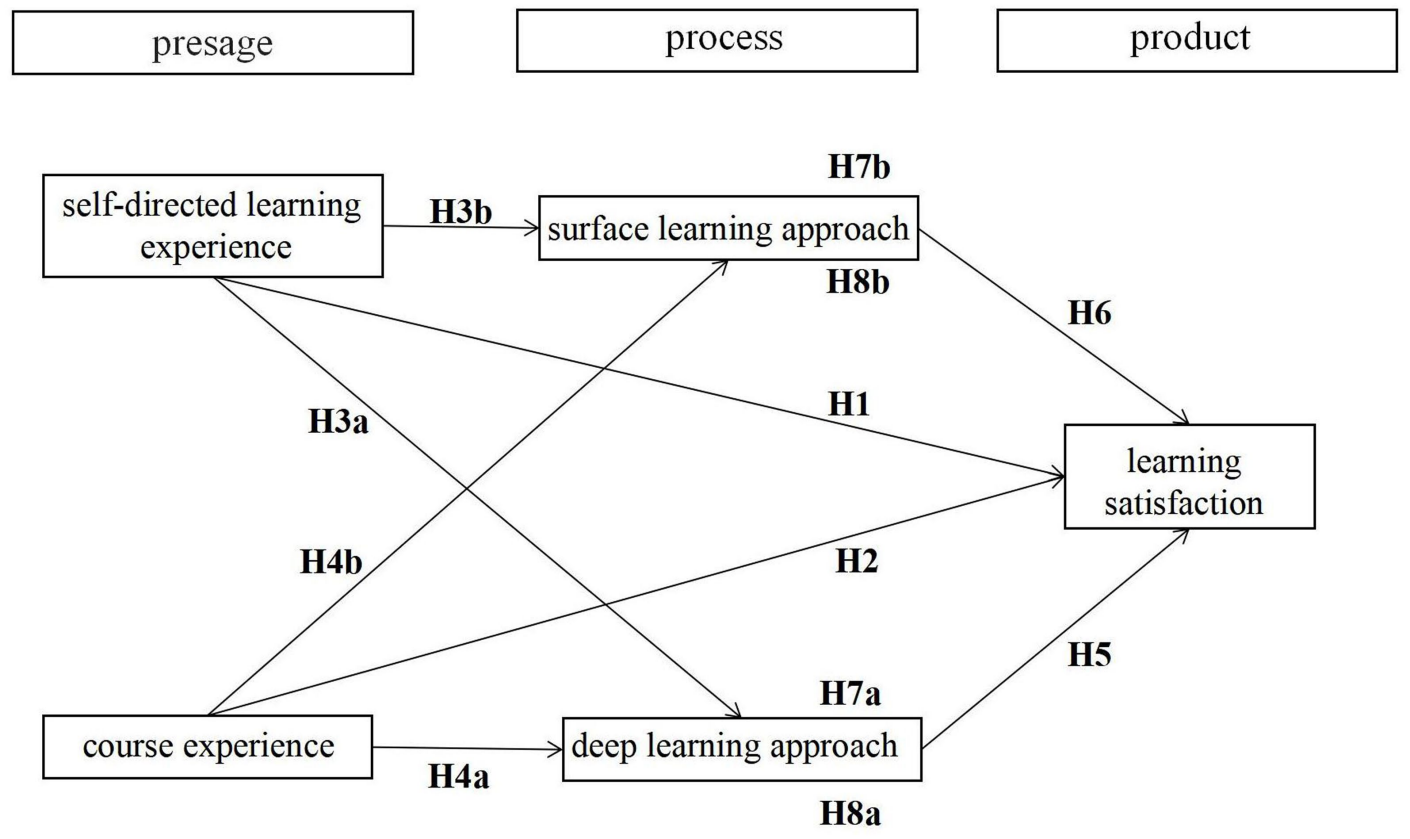
Hypothetical model.

This study investigates the mechanisms and processes of blended learning among students from diverse knowledge domains in the context of Chinese universities. Specifically, it explores the relationships among self-directed learning experience, course experience, the deep learning approach, the surface learning approach, and learning satisfaction. Considering that blended learning has become one of the core educational reforms in universities globally post-pandemic, it is crucial to understand students’ various learning experience in blended learning and how these experience are interconnected with other factors within the learning process theory framework. This research topic is worth investigating as the findings can contribute not only to further the development of blended learning theory but also to assist blended learning providers in delivering targeted interventions to enhance students’ learning satisfaction. The hypothesized model is depicted in Figure 2.

## 2 Materials and methods

### 2.1 Participants

The participants were from 4 universities located in Xuzhou, a second-tier city in China. The sample was selected by simple and random sampling. First, we got a list of first-year students in each university and allocated a unique identification number for each student. Then, we selected students corresponding to the size of the target sample according to a random way to ensure that each student had an opportunity to be selected. All of the samples were involved in at least one blended course. Questionnaires were distributed to 965 first-grade students, and 26 invalid questionnaires were eliminated due to incomplete responses. In the end, 939 valid questionnaires were collected, with a response rate of 97.306%. Among the survey respondents were 444 male students and 495 female students. The average age of the participants was 18.622 (SD = 0.601, range 17.030–21.011). There were 444 male students (mean age = 18.689, SD = 0.565) and 495 female students (mean age = 18.563, SD = 0.626). The distribution of participants by gender ratio was relatively balanced. Participants came from more than 20 disciplines, including humanities, education, science, and engineering.

### 2.2 Context and procedure

The courses that adopted the blended learning model had an average of three classes perweek (40 min per class). These courses are general education courses for the first semester of the first year of Chinese universities. After the courses, students completed all surveys which measured the students’ blended learning for these courses through an online platform called “Wen Juanxing.” They were informed that they could choose not to participate in the survey if they did not want to participate. Even if the participants chose not to participate in the survey, it would not affect their course grades or future opportunities. The answers are kept confidential and anonymous. Filling out the questionnaire took participants about 25 min.

### 2.3 Measures

#### 2.3.1 Self-rating scale of blended self-directed learning

Self-rating scale of blended self-directed learning (SRSBSDL), a modified version of Self-Rating Scale of Self-Directed Learning (SRSSDL), was adopted to evaluate students’ self-directed learning experience in the blended learning environment by measuring five dimensions of awareness (6 items), learning strategies (3 items), learning activities (4 items), evaluation (6 items), and interpersonal skills (6 items) (Williamson, 2007). Based on the SRSSDL, the content relating to blended learning was added to the SRSBSDL. For example, the original question “I consider teachers as facilitators of learning rather than providing information only” was rephrased as “I consider teachers as facilitators of learning rather than providing information only in blended learning.” The scale was answered on a 6-point Likert-type scale (ranging from “Never” to “Always”). The higher the scale’s score, the stronger the self-directed learning experience in the blended learning environment. The Cronbach’s α coefficient for SRSBSDL was 0.957.

#### 2.3.2 Blended course experience questionnaire

A modified version of Course Experience Questionnaire (CEQ) was adopted to measure students’ experience with blended courses from four dimensions of good teaching (6 items), generic skills (6 items), clear goals and standard (4 items), and overall satisfaction (1 items) (Curtis and Keeves, 2000). Based on the CEQ, the content relating to the blended course was added to the Blended course experience questionnaire (BCEQ). For example, the original question “The teacher motivated me to do my best work” was rephrased as “The teaching staff of the blended courses motivated me to do my best work when I am in blended learning.” The scale was answered on a 5-point Likert-type scale (ranging from “Strongly disagree” to “Strongly agree”). The higher the questionnaire’s score, the better the experience of students in blended learning. The Cronbach’s α coefficient for the scale was 0.961.

#### 2.3.3 Questionnaire of deep approach to blended learning

Three items were selected from the deep approach dimension of Revised Study Process Questionnaire (R-SPQ-2F) to form questionnaire of deep approach to blended learning (QDA-BL) (Biggs et al., 2001). Based on the R-SPQ-2F, the content relating to blended learning was added to QDA-BL. For example, the original question, “I feel that virtually any topic can be highly interesting once I get into it.” was rephrased as “I feel that virtually any topic can be highly interesting once I get into it in the blended learning environment.” The scale was answered on a 7-point Likert-type scale (ranging from “Never” to “Always”). The higher the students’ questionnaire scores, the more inclined they were to adopt a deep learning approach in a blended learning environment. The Cronbach’s α coefficient for the questionnaire was 0.875.

#### 2.3.4 Questionnaire of surface approach to blended learning

Three items were selected from the surface approach dimension of Revised Study Process Questionnaire (R-SPQ-2F) to form questionnaire of surface approach to blended learning (QSA-BL). Based on the R-SPQ-2F, the content relating to blended learning was added to QSA-BL. For example, the original question, “My aim is to pass the course while doing as little work as possible,” was rephrased as “My aim is to pass blended courses while doing as little work as possible.” The scale was answered on a 5-point Likert-type scale (ranging from “Never” to “Always”). The higher the students’ questionnaire scores, the more inclined they were to adopt a surface learning approach in a blended learning environment. The Cronbach’s α coefficient for the questionnaire was 0.782. The details of SRSBSDL, BCEQ, QDA-BL, and QSA-BL can be observed in Supplementary Appendix 1.

#### 2.3.5 Blended learning satisfaction

In this study, the score of learning satisfaction was obtained according to the students’ self-filled questionnaire. Students rated their satisfaction on a scale of 0 to 100, with higher scores representing higher learning satisfaction for these blended courses. The mean score of learning satisfaction was 74.196, and the standard deviation was 13.944. Unlike the other four measures assumed as latent variables, learning satisfaction was treated as an observed variable.

### 2.4 Data statistical analysis

The hypothetical research model was a multiple mediation model, where the deep or surface learning approach mediated the influence of self-directed learning experience and course experience on learning satisfaction. First of all, the common method deviation test was carried out. Then SPSS 22.0 was used for correlation analysis, reliability tests, and descriptive statistical analysis. The correlation analysis helps to understand the degree and direction of the correlation between variables and serves as the basis for calculating the discriminant validity (DV) of measurement models. The internal consistency reliability (ICR), convergent validity (CV), and DV were tested afterward. Subsequently, because the covariance-based structural equation modeling (CB-SEM) is suitable for confirmatory research with a theoretical basis, a CB-SEM was built to test Biggs’ learning process theory in the blended learning context (Hair et al., 2017; Rigdon et al., 2017). Finally, the mediating effects were analyzed by the bias-corrected percentile bootstrap method, which can be used to explore mediation models for large, medium, and small samples and estimate more accurate mediating effect size confidence intervals (MacKinnon et al., 2004). The analysis of the model and the mediation effects were conducted in the visual AMOS 26 software with full information maximum likelihood (FIML) (Graham, 2003).

## 3 Results

### 3.1 Test of common method bias

Harman’ s single-factor test was used to assess common method bias before formal data analysis (Podsakoff, 2003). All observed variables in this study were loaded into exploratory factor analysis (EFA) to determine whether the first factor accounted for most of the variance of the variable. The results showed that the first extraction factor explained 36.383% of the variance (less than 50%), indicating that the common method bias was not serious (Podsakoff and Organ, 1986).

In addition, confirmatory factor analysis (CFA) was also conducted to calculate the common method variance. All dimensions of the four latent variables (self-directed learning experience, course experience, the deep learning approach, and the surface learning approach) and one manifest variable (learning satisfaction) were included in the analysis of single-factor and five-factor confirmatory factors. Then, the goodness of fit indices of the single-factor and five-factor models were compared (see Table 1). The results showed that there was a significant difference between the five-factor model (*χ*^2^ = 454.443, df = 95) and the single-factor model (*χ*^2^ = 3769.703, df = 104), △*χ*^2^ = 3315.26, △df = 9, *p* < 0.001, supporting that the common methods variance does not affect the standardized path coefficients and the structural model fit indices (Mossholder et al., 1998; Iverson and Maguire, 2000).

**Table 1:** Test for common method bias.

Model	*χ* ^2^	df	△*χ*^2^	△df	*p*
Single-factor	3769.703	104	3315.26	9	0.000
Five-factor	454.443	95			

***p < 0.000.

### 3.2 Descriptive statistics and correlation analysis

Table 2 presents the descriptive statistics and correlational analysis results for variables of self-directed learning experience, course experience, learning approaches, and learning satisfaction. The correlation analysis results indicated that there was a significant positive correlation between any two of the variables of the self-directed learning experience, the course experience, the deep approach, and the learning satisfaction. Furthermore, the surface learning approach was significantly negatively correlated with the other four variables.

**Table 2:** Correlation analysis and descriptive statistical results.

	M	SD	1	AW	EV	IS	LS	LA	2	GT	GS	CGS	Overall-CE	3	4	5
1 SDLE	3.487	0.875	1	0.926	0.883	0.904	0.833	0.851	0.432	0.288	0.469	0.422	0.330	0.659	−0.312	0.518
AW	3.336	1.041		1	0.769	0.788	0.747	0.724	0.370	0.225	0.418	0.365	0.281	0.600	−0.285	0.450
EV	3.340	0.877			1	0.712	0.678	0.713	0.394	0.281	0.420	0.381	0.274	0.588	−0.258	0.452
IS	3.968	1.043				1	0.713	0.703	0.438	0.303	0.465	0.423	0.364	0.635	−0.341	0.506
LS	3.374	0.929					1	0.670	0.404	0.279	0.431	0.392	0.315	0.566	−0.244	0.468
LA	3.298	1.014						1	0.293	0.183	0.324	0.298	0.213	0.496	−0.213	0.409
2 CE	3.707	0.656							1	0.908	0.940	0.895	0.706	0.631	−0.289	0.399
GT	3.775	0.666								1	0.760	0.732	0.591	0.502	−0.265	0.333
GS	3.640	0.789									1	0.790	0.614	0.611	−0.247	0.379
CGS	2.449	0.465										1	0.642	0.615	−0.280	0.387
Overall-CE	3.833	0.817											1	0.517	−0.260	0.293
3 DLA	4.116	1.293												1	−0.255	0.501
4 SLA	2.455	0.790													1	−0.284
5 LSAT	74.196	13.944														1

All data were significantly correlated. **p < 0.001. SDLE, self-directed learning experience; IS, Interpersonal Skills; LA, learning activity; LS, Learning Strategies; EV, Evaluation; AW, Awareness; CE, Course Experience; GT, Good Teaching; GS, Generic Skills; CGS, Clear Goals and Standard; Overall-CE, Overall Course Experience; DLA, Deep Learning Approach; SLA, Surface Learning Approach; LSAT, learning satisfaction.

### 3.3 Measurement model analysis

The measurement models were tested on ICR, CV, and DV. ICR was implemented to evaluate the consistency of results across all indicators, where the value of CR (composite reliability) and CA (Cronbach’s α) should be greater than 0.7 (Fornell and Larcker, 1981). Table 3 showed that each CR and CA was more than 0.7, indicating good ICR. CV is described as a condition for relating to the variable construct. It is declared ideal and good when the average variance extracted (AVE) is more than 0.5 (Wijaya et al., 2022). Table 3 also showed that all the measurement models observed also had good CV, with the lowest AVE value of the surface learning approach being 0.557.

**Table 3:** Factor loading, internal consistency reliability, and convergent validity statistics.

Construct	Indicator	Std. factor loading	Item reliability	ICR		Convergence validity
			SMC	CR	CA	AVE
SDLE	IS	0.862	0.743	0.929	0.957	0.723
	LS	0.820	0.672			
	LA	0.815	0.664			
	EV	0.843	0.711			
	AW	0.907	0.823			
CE	Overall-CE	0.705	0.497	0.901	0.961	0.695
	CGS	0.883	0.780			
	GS	0.895	0.801			
	GT	0.839	0.704			
DLA	Q1	0.881	0.776	0.882	0.875	0.716
	Q2	0.936	0.876			
	Q3	0.705	0.497			
SLA	Q1	0.744	0.554	0.788	0.782	0.557
	Q2	0.846	0.716			
	Q3	0.633	0.401			

Table 4 showed that the entire AVE square root of the latent variable was larger than the correlation coefficient of other determinants, verifying that DV of this analysis was good (Fornell and Larcker, 1981). Hair et al. (2006) also proposed to observe the value of HTMT (Heterotrait-Monotrait ratio of correlations) to highly analyze DV specifically. DV is considered good when the HTMT value does not exceed the 0.9 threshold. Table 5 demonstrated that the highest HTMT value was 0.725 (SDLE-DLA), proving the DV between the measurement models was good.

**Table 4:** Fornell-Larcker test for discriminant validity.

	AVE	SDLE	CE	DLA	SLA
SDLE	**0.723**	**0.850**			
CE	**0.695**	0.370	**0.834**		
DLA	**0.716**	0.659	0.631	**0.846**	
SLA	**0.557**	−0.312	−0.289	−0.255	**0.746**

AVE = 0.723, 0.695, 0.716 AND 0.557; $\sqrt{\mathrm{AVE}}$ = 0.850, 0.834, 0.846, 0.746.

**Table 5:** Heterotrait-Monotrait test for discriminant validity.

	SDLE	CE	DLA	SLA
SDLE				
CE	0.471			
DLA	0.725	0.723		
SLA	0.357	0.359	0.308	
LSAT	0.538	0.420	0.535	0.322

### 3.4 Structural equation modeling analysis

A full structural equation model was employed to test our hypothetical model. The C.R. of multivariate value was 63.760, indicating significant multivariate non-normality in the data. As a result, the Bollen-Stine bootstrap *p* procedure was used to adjust model fit and parameter estimates to accommodate the lack of multivariate normality (Bollen and Stine, 1992). It was found that Bollen-Stine bootstrap *p* = 0.000, showing that the expansion of the *χ*^2^ is due to the large sample size. After the adjustment, the model had good indices with the data, i.e., *χ*^2^/df = 1.283, CFI = 0.997, GFI = 0.988, RMSEA = 0.017, SRMR = 0.041.

**Figure 3:**
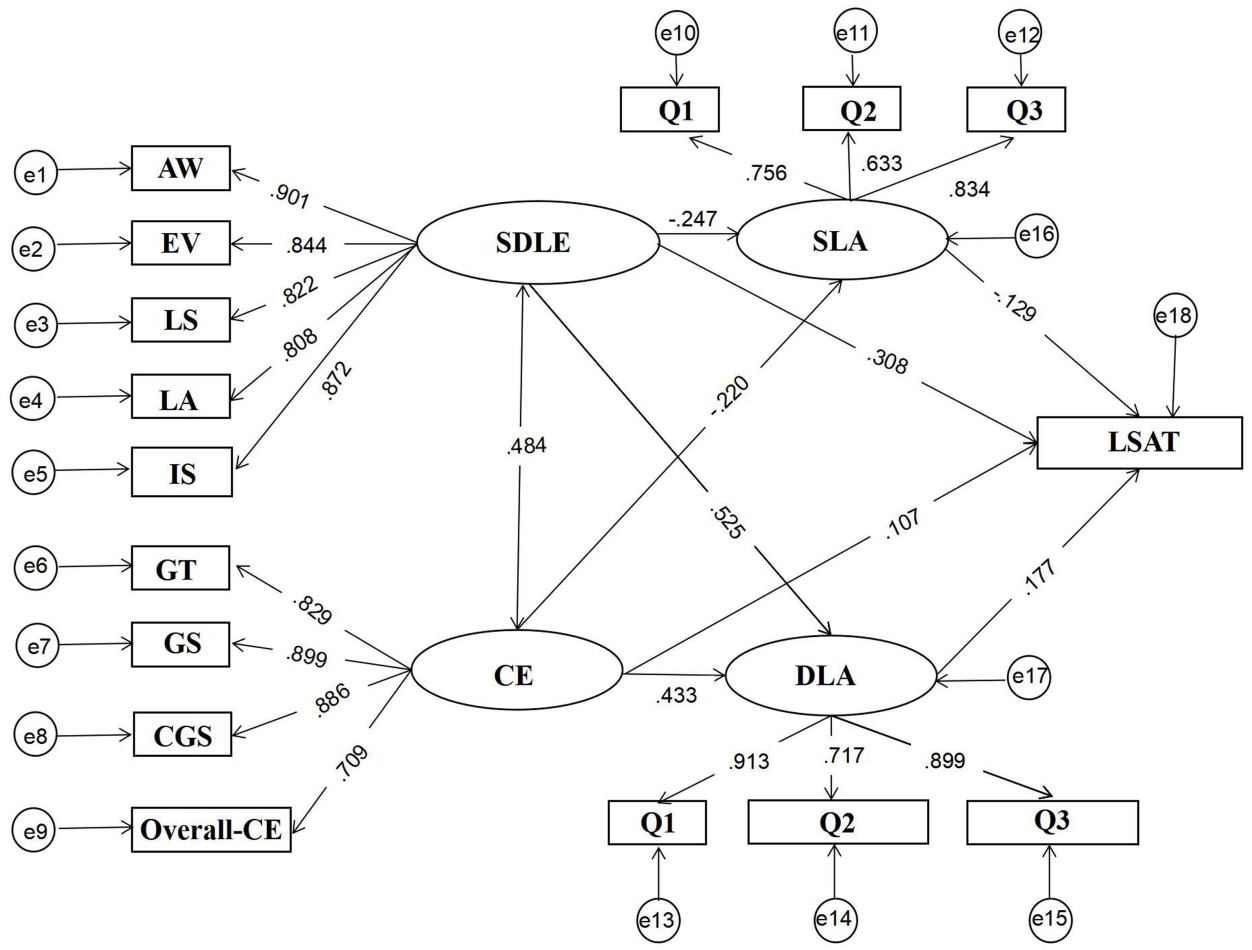
Mediation model effect plot.

The standardized path coefficients were presented in Figure 3. The results showed that self-directed learning experience had a positive direct effect on learning satisfaction (*β* = 0.308, *p* < 0.001) and course experience had a positive direct effect on learning satisfaction (*β* = 0.107, *p* < 0.05), which confirmed H1 and H2, that is, self-directed learning experience (H1) and course experience (H2) can directly predict learning satisfaction in Chinese university students’ learning.

In addition, self-directed learning experience had a positive effect on the deep learning approach (*β* = 0.525, *p* < 0.001) and a negative effect on the surface learning approach (*β* = −0.247, *p* < 0.001), which confirmed H3, that is, self-directed learning experience could positively predict the deep learning approach (H3a) and negatively predict the surface learning approach (H3b) in Chinese university students’ learning. Course experience had a positive effect on the deep learning approach (*β* = 0.433, *p* < 0.001) and had a negative effect on the surface learning approach (*β* = −0.220, *p* < 0.001), which confirmed H4, that is, course experience can positively predict the deep learning approach (H4a) and negatively predict the surface learning approach (H4b) in Chinese university students’ learning.

Furthermore, the deep learning approach had a positive effect on learning satisfaction (*β* = 0.177, *p* < 0.01), which confirmed H5, that is, the deep learning approach can positively predict learning satisfaction in Chinese university students’ learning. The surface approach had a negative effect on learning satisfaction (*β* = −0.129, *p* < 0.001), which confirmed H6, that is, the surface learning approach can negatively predict the learning satisfaction in Chinese university students’ learning.

### 3.5 Mediation effect analysis

The bootstrapping text in SEM was further used to examine the mediating effect of learning approaches (the sampling frequency was 2,000). According to Shrout and Bolger, when the 95% confidence interval (CI) of the indirect effect contains zero, the mediating effect is not significant; when neither the 95% CI of the indirect effect nor the 95% CI of the direct effect contains zero, the mediating effect is significant and is a partial mediating effect; when the 95% CI of the indirect effect does not contain zero, and the 95% CI of the direct effect contains zero, the mediating effect is significant and is a direct mediating effect (Shrout and Bolger, 2002).

From Table 6, the results indicated that on the path from the self-directed learning experience to the learning satisfaction, the direct effect value was 0.966 and the mediating effect value was 0.391 (0.291 + 0.100). More specifically, for the mediating effect produced by path one (self-directed learning experience → deep learning approach → learning satisfaction), the indirect effect was 0.291, and for path two (self-directed learning experience → surface learning approach → learning satisfaction), the indirect effect was 0.100. The results indicated that the bias-corrected 95% and percentile 95% CI of path one were [0.102, 0.487] and [0.100, 0.486], respectively, indicating that the mediating effect of the deep learning approach was significant. Thus, the findings supported H7a, which held that “the effect of self-directed learning experience on learning satisfaction is mediated by the deep learning approach.” In addition, the bias-corrected 95% CI and percentile 95% CI [0.047, 0.168] of path two suggested that the mediation effect of the surface learning approach was significant. These findings supported H7b, which held that “self-directed learning experience on learning satisfaction is mediated by the surface learning approach.” More importantly, the bias-correction and percentile 95% CI of the direct effect were [0.674, 1.255] and [0.674, 1.254], which do not contain zero, indicating that self-directed learning experience on learning satisfaction was partially mediated by the deep and surface learning approach.

**Table 6:** The analysis of the mediating effect of self-directed learning experience to learning satisfaction.

Mediating effect	Point estimates	Product of coefficient		Bootstrapping			
				Bias-corrected 95% CI		Percentile 95% CI	
		SE	Z	Lower	Upper	Lower	Upper
Indirect effect 1							
Path one							
Self-directed learning experience →deep learning approach →learning satisfaction	0.291	0.098	2.97	0.102	0.487	0.100	0.486
Indirect effect 2							
Path two							
Self-directed learning experience→ surface learning approach→ learning satisfaction	0.100	0.031	3.23	0.047	0.168	0.047	0.168
Direct effect							
Self-directed learning experience→ learning satisfaction	0.966	0.147	6.571	0.674	1.255	0.674	1.254

On the path from course experience to learning satisfaction, the direct effect value was 2.585 and the mediating effect value was 2.527 (1.844 + 0.683). Specifically, for the mediating effect produced by path one (course experience → deep learning approach → learning satisfaction), the indirect effect was 1.844, and for that produced by path two (course experience → surface learning approach → learning satisfaction), the indirect effect was 0.683 (see Table 7). The bias-corrected 95% [0.606, 3.130] and percentile 95% CI [0.612, 3.158] of path one indicated that the mediating effect of the deep learning approach was significant, thus supporting H8a, which held that “the effect of course experience on learning satisfaction is mediated by the deep learning approach.” In addition, the bias-corrected [0.301, 1.172] and percentile 95% CI [0.299, 1.161] of path two suggested that the surface approach had a significant mediation effect, thus supporting H8b, which held that “the effect of course experience on learning satisfaction is mediated by the surface learning approach.” Furthermore, the bias-corrected and percentile 95% CI of the direct effect were [0.653, 4.822] and [0.482, 4.703], which do not contain zero, indicating that the effect of course experience on learning satisfaction was partially mediated by the deep learning approach and the surface learning approach (see Table 7).

**Table 7:** The analysis of the mediating effect of course experience to learning satisfaction.

Mediating effect	Point estimates	Product of coefficient		Bootstrapping			
				Bias-corrected 95% CI		Percentile 95% CI	
		SE	Z	Lower	Upper	Lower	Upper
Indirect effect 1							
Path one							
Course experience→ deep learning approach→ learning satisfaction	1.844	0.624	2.956	0.606	3.130	0.612	3.158
Indirect effect 2							
Path two							
Course experience→ surface learning approach→ learning satisfaction	0.683	0.223	3.063	0.301	1.172	0.299	1.161
Direct effect							
Course experience→ learning satisfaction	2.585	1.064	2.430	0.653	4.822	0.482	4.703

## 4 Discussion

This study investigated learning satisfaction of university students in a blended learning environment and explored it’s influencing factors. Based on Biggs’ learning process theory, data were collected through a questionnaire survey, and a structural equation model was employed to examine the predictive effect of students’ learning experience on learning satisfaction and the mediating role of learning approaches. The results indicates that self-directed learning experience and course experience in blended learning can directly predict university students’ learning satisfaction, with the deep and surface learning approaches partially mediating the relationship. These findings highlight the significance of blended learning experience and learning approaches on learning satisfaction and can guide educators to better leverage the role of blended instruction.

### 4.1 The overall situation regarding learning experience, learning approaches, and learning satisfaction

The score of self-directed learning experience is at the average level. Scores of course experience and learning satisfaction are all rated above average. It means that students’ course experience is better than the self-directed learning experience, and the blended learning environment contributes to students’ classroom learning to improve their overall satisfaction with their studies. The score for the deep learning approach is average, while the score for the surface learning approach is below average, which suggests that in a blended learning environment, students tend to prefer deep learning strategies that facilitate a deeper understanding and acquisition of knowledge and skills compared to the surface learning approach. However, there is room for further improvement in using deep learning strategies, and some students may require more support and guidance to avoid relying on less effective surface learning approaches.

Regarding self-directed learning experience dimensions, interpersonal skills received the highest score. In contrast, learning activities received the lowest score, which aligns with the findings of Hwang and Kim in an online learning environment (Hwang and Kim, 2023). This indicates that interpersonal competence is crucial for students’ self-directed learning, whether in an online or blended learning environment. The two learning environments require interactions between students and teachers or peers, making interpersonal interaction an essential aspect of self-directed learning (Bouilheres et al., 2020; Hadiyanto et al., 2021). The social cognitive theory suggests that students’ learning process and outcomes are not only determined by their individual cognitive processes and knowledge structures but are the result of complex processes in which students interact with their surroundings and society (Bandura, 2002; Schunk and DiBenedetto, 2020). In a blended learning environment, when students communicate with others, they receive feedback and support from fellow students and instructors, deepening their understanding of the learning tasks and knowledge acquisition. Moreover, interpersonal skills can stimulate collaborative learning, transforming learning environment with information and communication technology to help students build higher knowledge in blended learning (Zhao et al., 2022). The lower score for learning activities indicates that learning activities are one of the factors that restrict students’ self-directed learning. Students may need more ability to select learning activities that suit their needs or encounter difficulties while implementing learning activities (Dakhi et al., 2020; Sitthiworachart et al., 2021; Syahrawati et al., 2022).

In the aspect of course experience, the highest-rated dimension is good teaching, followed by general skills and clear goals and standard. These results differ from the findings of Diseth, where general skills received the highest score in their study. One possible reason for this discrepancy could be the difference in the courses. Diseth’ s study focused on a specialized psychology course, which likely emphasized the development of students’ professional skills and knowledge (Altman, 1996; Diseth, 2007). Therefore, teachers of this course may pay more attention to improving students’ skills in applying knowledge, so students will get the higher score in general skills. In contrast, the blended courses in this study are general education courses with comprehensive, extensive, basic, and cross-cultural characteristics. The teaching purposes of these courses are to impart basic knowledge, broaden horizons, and improve humanistic literacy in China (Li and Liu, 2023). Students scoring higher in the dimension of good teaching may be attributed to the effective guidance of teachers in achieving these purposes. In the present study, the lower score for general skills reflects that blended courses require higher teaching skills from instructors, and it is more challenging for them to attain the skill level that satisfies students. In addition to the instructional design and guidance skills required in traditional classrooms, instructors in a blended learning environment must possess technological literacy and proficiency in online teaching tools (De Vera et al., 2021). They should also be able to design and manage tasks in an online learning environment to stimulate students’ personalized learning needs (Akyol et al., 2009; King and Cerrone, 2012; Vaughan et al., 2013). This highlights the need for instructors to possess a diverse and complex set of skills. In addition, the low score of the clear goals and standard show that in the blended courses, students do not have a better grasp of learning objectives and standards because they have to deal with more complex learning tasks. Therefore, teachers need to communicate curriculum objectives and learning standards more clearly and conduct personalized teacher feedback more effectively to ensure that students can understand and accurately assess their own learning progress.

### 4.2 The direct effect of learning experience on learning satisfaction

Research shows that self-directed learning experience directly predicted students’ learning satisfaction in a blended learning environment. This finding supports previous studies on the relationship between individual factors and learning outcomes among students (Marsh et al., 2005; Jiang et al., 2022; Zhao et al., 2022; Hua and Wang, 2023), indicating that self-directed learning ability can help students better adapt to a blended learning environment and develop improved self-management and learning skills for future learning and work contexts. Additionally, course experience can also directly predict students’ learning satisfaction, highlighting the importance of enhancing course experience to improve students’ satisfaction with their academic performance. This conclusion is consistent with the findings of Arbaugh and Duray (2002), So and Brush (2008), Sun et al. (2008), and Diep et al. (2017), and Guo et al. (2017). Students face more challenging course content and intensive learning tasks in a blended learning environment. If their course experience is terrible, students may quickly lose motivation, impacting their learning effectiveness and learning satisfaction. These research findings align with the self-determination theory proposed, suggesting that individuals’ satisfaction is associated with self-directed and perceived interpersonal support (Black and Deci, 2000). In a blended learning environment, when learners have sufficient autonomy to choose their learning content and control their learning methods and pace, and when they engage in social, collaborative activities with peers, receiving positive, supportive feedback and rewards from instructors, their intrinsic learning motivation and learning satisfaction tend to be higher. However, it should be noted that for learning satisfaction, the impact of self-directed learning experience is more significant than that of course experience. This result differs from the findings of Sun et al. (2008) and Diep et al. (2017). The two studies indicated that the course quality experience had a more significant influence on satisfaction than individual factors. The reason for the inconsistency between the results of this study and those of Sun et al. may lie in the fact that Sun’s study was conducted in an online learning environment. Sun suggests that in an online learning environment, there is relatively less interaction between students and their peers or instructors (Sun et al., 2008). Thus, students’ course experience relies more on the design and quality of the online course itself rather than individual factors such as learning ability or study habits. In Diep’s study, factors related to the course experience, such as teachers’ expertise and students’ perceived task value, directly and significantly predicted learning satisfaction. In contrast, self-efficacy, an individual factor, did not directly predict learning satisfaction (Diep et al., 2017). This discrepancy may be attributed to differences in the courses’ nature and the participants’ age within the samples.

### 4.3 The mediating effect of learning approaches

The results indicate that in a blended learning environment, university students’ self-directed learning experience and course experience directly predict learning satisfaction and indirectly predict it through the deep learning and the surface learning approaches. This can be explained by Csikszentmihalyi’s theory of flow experience (Csikszentmihalyi, 1988). The concept of “flow” experience refers to a state in which individuals fully engage their abilities and skills to tackle challenging yet controllable learning tasks while experiencing clear goals and accurate feedback in the course (Nakamura and Csikszentmihalyi, 2009). This state allows them to transcend temporal and spatial constraints, forget about themselves, and attain a transcendent experience of self (Abuhamdeh, 2020). In a blended learning environment supported by technology, if online learning resources and social platforms can provide students with opportunities for self-directed learning at different times and locations while also providing effective feedback and support, students can effectively utilize these resources to experience high-quality learning experience, achieve better outcomes in deep learning, and consequently improve their learning satisfaction. Conversely, if there is a lack of effective feedback, a mismatch between the difficulty of the learning task and the learners’ skill level, or if the course design is unappealing, students may adopt the surface learning approach where they focus solely on achieving the minimum requirements and exam scores without paying attention to the intrinsic understanding and application of knowledge. This learning approach may decrease students’ learning satisfaction as the memorized knowledge from surface learning is quickly forgotten and cannot be effectively applied in real-life contexts.

It is worth noting that, both in terms of learning approaches and learning satisfaction, the influence of self-directed learning experience was greater than that of course experience. This suggests that the self-directed learning experience substantially impacts students’ subsequent learning process and outcomes in a blended learning environment more than course experience. This could be because, in contrast to traditional teaching methods that rely more on teaching quality and course design, students in a blended learning environment are no longer solely dependent on teachers’ instructions. Instead, they independently choose learning resources and approaches that suit their needs and interests, managing their learning process (Geng et al., 2019). This self-directed learning experience, characterized by independent exploration, proactive problem-solving, self-reflection, and continuous adjustment, enhances students’ engagement and motivation throughout the learning process, leading to long-term learning outcomes (Shen et al., 2013; Lee et al., 2014). This reflects the need for more personalized, student-centered, self-directed, and inquiry-based learning in a blended learning environment. However, it is essential to clarify that course experience is not insignificant, as obtaining a good course experience is also essential for generating positive learning outcomes (So and Brush, 2008; Sun et al., 2008; Guo et al., 2017). Students need clear and well-designed courses to guide their learning direction and help them better master self-directed learning skills.

## 5 Implications of the study

The level of engagement and learning strategies in university students’ self-directed learning activities still need further enhancement. Teachers can create a positive learning environment through interactive learning platforms and tools, design diverse blended learning activities, and establish positive motivation and reward mechanisms to stimulate students’ interest and motivation to participate in activities. Additionally, teachers must explicitly teach students how to develop blended learning plans, set learning goals, allocate time resources effectively, and manage online and offline tasks strategically and skillfully, enabling them to take control of the learning process. Teachers can also encourage students to prioritize and schedule learning activities, analyze their learning strategies, identify strengths and areas for improvement, and promote regular reflection and self-assessment of the learning process through guided questioning, writing, or discussions.

Improving university students’ positive teaching experience is also necessary. Blended learning requires an effective combination of online and face-to-face instruction, so teachers should adequately prepare and plan course content, learning activities, and resources to ensure a smooth and organized teaching process. As blended learning provides opportunities for diverse teaching methods and resources, teachers can employ various instructional approaches, such as lectures, case studies, group discussions, practical tasks, simulated experiments, and multimedia materials to cater to students’ different learning needs and styles. Teachers can also organize remote collaborative learning among students, such as collaborative document editing and multimedia project creation, to enhance the collective experience among students.

Regarding learning approaches, it is essential to note that being a deep or surface learner is not a permanent characteristic of students. The type of learning approach students pursue largely depends on their previous academic experience and the nature of educational tasks. Therefore, on the one hand, teachers need to promote the adoption of deep learning methods by improving students’ learning experience in blended learning environments. Specifically, teachers can help students establish clear, challenging, and measurable goals and provide relevant guidance and support to help them progress in deep learning. Teachers can also encourage students to develop meta-cognitive awareness and provide meta-cognitive support to facilitate deep understanding. On the other hand, regarding the issue of college students adopting surface learning methods, it is necessary to address their self-directed learning experience and improve their learning motivation and strategies to avoid adopting surface learning approaches. More personalized support and guidance should be provided for students with more evident surface learning tendencies to help them overcome the influence of surface learning approaches and achieve better learning outcomes.

## 6 Conclusion

Through the lens of Biggs’ learning process theory, this study has investigated the relationships between Chinese university students’ learning experience and learning satisfaction and the mediating mechanisms between the two. The results have indicated that self-directed learning experience and course experience directly predict students’ learning satisfaction. Different learning approaches, namely deep and surface learning, mediate the relationships between learning experience and learning satisfaction.

This paper contributes to the existing literature in three ways. Firstly, it captures the new learning characteristics of university student groups in blended courses and reveals how their learning experience, mediated by learning approaches, impacts learning satisfaction within blended learning environments. Secondly, while previous research has mainly focused on the technological and instructional features of blended learning (Miyazoe and Anderson, 2010; Korr et al., 2012; Keengwe and Kang, 2013; Xiao, 2016; Feng et al., 2018), this study emphasizes the learning behaviors and psychology of students in blended learning environments, which have previously been overlooked, thus further advancing research on blended learning from the learner’s perspective. Moreover, this paper integrates the self-learning characteristic of blended learning into the framework of learning process theory, providing a theoretical analysis framework specifically applicable to blended learning. Lastly, this study provides empirical evidence and practical recommendations for emerging blended teaching practices in the post-pandemic era. This advances the understanding of how teaching and learning support at policy levels should be tailored for university students within blended learning environments.

## 7 Limitations and future directions

The following limitations need to be pointed out. To begin with, the sample in this study consisted of Chinese first-year university students, which may limit the generalizability of the research findings on blended learning mechanisms to other sociocultural contexts and students at different academic levels. Future explorations should include samples from diverse sociocultural backgrounds and different academic levels to validate the results of this study. Secondly, merely two perspectives of presage factors (self-directed learning experience and course experience) are included as the independent variables. However, according to Biggs’ learning process theory, the presage factors include other aspects, such as prior knowledge and motivation (Biggs, 1993). These variables may jointly influence learning outcomes with learning experience (Gentrup et al., 2020; Murphy et al., 2020; Brod, 2021). Future research should combine these variables to test the adaptability of Biggs’ learning process theory model in blended learning environments. Thirdly, Biggs’ learning process theory discusses that previous learning outcomes will further shape the presage factors for future learning, thereby exerting new influences on subsequent learning process (Biggs, 1993; Biggs et al., 2001). However, this study employed a cross-sectional design, which limits causal and directional assumptions. This reminds us that learning satisfaction of first-year students may be more strongly influenced by their learning approaches and experience before university. Their learning experience and approaches may also change over time. Therefore, future longitudinal studies can be conducted to investigate further the dynamic relationships between learning experience, learning approaches, and learning outcomes, thereby strengthening the argument for causal relationships among the three variables. Finally, the blended courses involved in this study are general education courses, which have some common features but also have their own unique characteristics. Therefore, future studies should focus on one specific blended course to explore the blended learning mechanism unique to it in depth.

## Data availability statement

The raw data supporting the conclusions of this article will be made available by the authors, without undue reservation.

## Ethics statement

The studies involving humans were approved by the Xuzhou Kindergarten Teachers College’s Academic Committee. The studies were conducted in accordance with the local legislation and institutional requirements. The participants provided their written informed consent to participate in this study.

## Author contributions

MH: Conceptualization, Methodology, Resources, Writing – original draft. LW: Conceptualization, Formal analysis, Methodology, Writing – review & editing. JL: Data curation, Writing – review & editing.

## References

[ref1] Abraham A. (2006). “Teaching and learning in accounting education: students’ perceptions of the linkages between teaching context, approaches to learning and outcomes” in Celebrating accounting. eds. Juchau R. Tibbits G. (Sydney: University of Western Sydney), 1–15.

[ref2] Abuhamdeh S. (2020). Investigating the “flow” experience: key conceptual and operational issues. Front. Psychol. 11:158. doi: 10.3389/fpsyg.2020.00158, PMID: 32116954 PMC7033418

[ref3] Akyol Z. Garrison D. R. Ozden M. Y. (2009). Development of a community of inquiry in online and blended learning contexts. Proc. Soc. Behav. Sci. 1, 1834–1838. doi: 10.1016/j.sbspro.2009.01.324

[ref4] Al Awamleh A. (2020). Students satisfaction on blended learning in the school of sport sciences. Ann. Appl. Sport. Sci. 8:e803. doi: 10.29252/aassjournal.803

[ref5] Altman I. (1996). Higher education and psychology in the millennium. Am. Psychol. 51, 371–378. doi: 10.1037/0003-066X.51.4.371

[ref6] Arbaugh J. B. (2000). Virtual classroom characteristics and student satisfaction with internet-based MBA courses. J. Manag. Educ. 24, 32–54. doi: 10.1177/105256290002400104

[ref7] Arbaugh J. B. Duray R. (2002). Technological and structural characteristics, student learning and satisfaction with web-based courses: an exploratory study of two on-line MBA programs. Manag. Learn. 33, 331–347. doi: 10.1177/1350507602333003

[ref8] Asghar M. Z. Iqbal A. Seitamaa-Hakkarainen P. Barbera E. (2021). Breaching learners’ social distancing through social media during the COVID-19 pandemic. Int. J. Environ. Res. Public Health 18:11012. doi: 10.3390/ijerph182111012, PMID: 34769534 PMC8583489

[ref9] Bandura A. (2002). Social cognitive theory in cultural context. Appl. Psychol. 51, 269–290. doi: 10.1111/1464-0597.00092

[ref10] Banerjee G. (2011). Blended environments: learning effectiveness and student satisfaction at a small college in transition. J. Async. Learn. Netw. 15, 8–19. doi: 10.24059/olj.v15i1.190

[ref11] Batista-Toledo S. Gavilan D. (2023). Student experience, satisfaction and commitment in blended learning: a structural equation modelling approach. Mathematics 11:749. doi: 10.3390/math11030749

[ref12] Benson P. (2013). Teaching and researching autonomy 4th ed. London: Routledge.

[ref13] Biggs J. B. (1989). Approaches to the enhancement of tertiary teaching. High. Educ. Res. Dev. 8, 7–25. doi: 10.1080/0729436890080102

[ref14] Biggs J. B. (1993). What do inventories of students’ learning processes really measure? A theoretical review and clarification. Br. J. Educ. Psychol. 63, 3–19. doi: 10.1111/j.2044-8279.1993.tb01038.x 8466833

[ref15] Biggs J. B. Kember D. Leung D. Y. (2001). The revised two-factor study process questionnaire: R-SPQ-2F. Br. J. Educ. Psychol. 71, 133–149. doi: 10.1348/000709901158433, PMID: 11307705

[ref16] Black A. E. Deci E. L. (2000). The effects of instructors’ autonomy support and students’ autonomous motivation on learning organic chemistry: a self-determination theory perspective. Sci. Educ. 84, 740–756. doi: 10.1002/1098-237X(200011)84:6<740::AID-SCE4>3.0.CO;2-3

[ref17] Bobe B. J. Cooper B. J. (2019). The effect of language proficiency on approaches to learning and satisfaction of undergraduate accounting students. Account. Educ. 28, 149–171. doi: 10.1080/09639284.2017.1396481

[ref18] Bobe B. J. Cooper B. J. (2020). Accounting students’ perceptions of effective teaching and approaches to learning: impact on overall student satisfaction. Account. Finance 60, 2099–2143. doi: 10.1111/acfi.12364

[ref19] Bollen K. A. Stine R. A. (1992). Bootstrapping goodness-of-fit measures in structural equation models. Sociol. Methods Res. 21, 205–229. doi: 10.1177/0049124192021002004

[ref20] Bonk C. J. Graham C. R. (2012). The handbook of blended learning: global perspectives, local designs. San Francisco, CA: John Wiley & Sons.

[ref21] Bouilheres F. Le L. T. V. H. McDonald S. Nkhoma C. Jandug-Montera L. (2020). Defining student learning experience through blended learning. Educ. Inf. Technol. 25, 3049–3069. doi: 10.1007/s10639-020-10100-y

[ref22] Brod G. (2021). Toward an understanding of when prior knowledge helps or hinders learning. Npj. Sci. Learn. 6:24. doi: 10.1038/s41539-021-00103-w, PMID: 34413307 PMC8377096

[ref23] Cheon J. Lee S. Crooks S. M. Song J. (2012). An investigation of mobile learning readiness in higher education based on the theory of planned behavior. Comput. Educ. 59, 1054–1064. doi: 10.1016/j.compedu.2012.04.015

[ref24] Collins A. Brown J. S. Newman S. E. (2018). “Cognitive apprenticeship: teaching the crafts of reading, writing, and mathematics” in Knowing, learning, and instruction. ed. Resnick L. B. (New York, NY: The Erlbaum press), 453, 494.

[ref25] Collis B. Moonen J. (2012). Flexible learning in a digital world: experiences and expectations. London: Taylor & Francis Group.

[ref26] Csikszentmihalyi M. (1988). The flow experience and its significance for human psychology. Optim. Exper. Psychol. Stud. Flow. Conscious. 2, 15–35. doi: 10.1017/CBO9780511621956.002

[ref27] Curtis D. D. Keeves J. P. (2000). The course experience questionnaire as an institutional performance indicator. Int. Educ. J. 1, 73–82.

[ref28] Dakhi O. Jama J. Irfan D. (2020). Blended learning: a 21st century learning model at college. Int. J. Multi Sci. 1, 50–65.

[ref29] de la Fuente J. D. L. Sander P. Kauffman D. F. Yilmaz M. (2020). Differential effects of self-vs. external-regulation on learning approaches, academic achievement, and satisfaction in undergraduate students. Front. Psychol. 11:543884. doi: 10.3389/fpsyg.2020.543884, PMID: 33117221 PMC7575817

[ref30] De Vera J. L. Andrada M. D. Bello A. De Vera M. G. (2021). Teachers’ competencies in educational technology integration on instructional methodologies in the new normal. Lukad: An. Online. J. Pedag. 1, 61–80.

[ref31] Diep A. N. Zhu C. Struyven K. Blieck Y. (2017). Who or what contributes to student satisfaction in different blended learning modalities? Br. J. Educ. Technol. 48, 473–489. doi: 10.1111/bjet.12431

[ref32] Diseth A. (2007). Approaches to learning: course experience and examination grade among undergraduate psychology students: testing of mediator effects and construct validity. Stud. High. Educ. 32, 373–388. doi: 10.1080/03075070701346949

[ref33] Diseth Å. Pallesen S. Brunborg G. S. Larsen S. (2010). Academic achievement among first semester undergraduate psychology students: the role of course experience, effort, motives and learning strategies. High. Educ. 59, 335–352. doi: 10.1007/s10734-009-9251-8

[ref34] Diseth A. Pallesen S. Hovland A. (2006). Course experience, approaches to learning and academic achievement. Educ. Train. 48, 156–169. doi: 10.1108/00400910610651782

[ref35] Donnelly R. (2010). Harmonizing technology with interaction in blended problem-based learning. Comput. Educ. 54, 350–359. doi: 10.1016/j.compedu.2009.08.012

[ref36] Feng X. Y. Wang R. X. Wu Y. J. (2018). A literature review on blended learning: based on analytical framework of blended learning (in Chinese). Dist. Educ. J. 36, 13–24. doi: 10.15881/j.cnki.cn33-1304/g4.2018.03.002

[ref37] Fornell C. Larcker D. F. (1981). Evaluating structural equation models with unobservable variables and measurement error. J. Mark. Res. 18, 39–50. doi: 10.1177/002224378101800104

[ref38] Ganotice J. Chan L. K. (2019). How can students succeed in computer-supported inter-professional team-based learning? Understanding the underlying psychological pathways using Biggs’ 3P model. Comput. Hum. Behav. 91, 211–219. doi: 10.1016/j.chb.2018.09.029

[ref39] Garrison D. R. Anderson T. Archer W. (2001). Critical thinking, cognitive presence, and computer conferencing in distance education. Am. J. Distance. Educ. 15, 7–23. doi: 10.1080/08923640109527071

[ref40] Garrison D. R. Vaughan N. D. (2013). Institutional change and leadership associated with blended learning innovation: two case studies. Internet High. Educ. 18, 24–28. doi: 10.1016/j.iheduc.2012.09.001

[ref41] Geng S. Law K. M. Niu B. (2019). Investigating self-directed learning and technology readiness in blending learning environment. Int. J. Educ. Technol. High. Educ. 16, 1–22. doi: 10.1186/s41239-019-0147-0

[ref42] Gentrup S. Lorenz G. Kristen C. Kogan I. (2020). Self-fulfilling prophecies in the classroom: teacher expectations, teacher feedback and student achievement. Learn. Instr. 66:101296. doi: 10.1016/j.learninstruc.2019.101296

[ref43] Graham J. W. (2003). Adding missing-data-relevant variables to FIML-based structural equation models. Struct. Equ. Modeling 10, 80–100. doi: 10.1207/S15328007SEM1001_4

[ref44] Guo J. Ji G. (2019). The relationship between college students’ perceptions of the learning environment and learning outcomes: the mediating role of student engagement. J. Psychol. Sci. 42, 868–875. doi: 10.16719/j.cnki.1671-6981.20190415

[ref45] Guo J. Yang L. Shi Q. (2017). Effects of perceptions of the learning environment and approaches to learning on Chinese undergraduates’ learning. Stud. Educ. Eval. 55, 125–134. doi: 10.1016/j.stueduc.2017.09.002

[ref46] Gurpinar E. Kulac E. Tetik C. Akdogan I. Mamakli S. (2013). Do learning approaches of medical students affect their satisfaction with problem-based learning? Adv. Physiol. Educ. 37, 85–88. doi: 10.1152/advan.00119.2012, PMID: 23471254

[ref47] Hadiyanto H. Failasofah F. Armiwati A. Abrar M. Thabran Y. (2021). Students’ practices of 21st century skills between conventional learning and blended learning. J. Unive. Teach. Learn. Pract. 18:7. doi: 10.53761/1.18.3.7

[ref48] Hair J. F. Black B. Babin B. Anderson R. E. Tatham R. L. (2006). Multivariate data analysis. 6th ed. New York: Prentice Hall.

[ref49] Hair J. F. Matthews L. M. Matthews R. L. Sarstedt M. (2017). PLS-SEM or CB-SEM: updated guidelines on which method to use. Int. J. Multivar Data Anal. 1, 107–123. doi: 10.1504/IJMDA.2017.087624

[ref50] Halverson L. R. Graham C. R. (2019). Learner engagement in blended learning environments: a conceptual framework. Online Learn. 23, 145–178. doi: 10.24059/olj.v23i2.1481

[ref51] Henrie C. R. Bodily R. Manwaring K. C. Graham C. R. (2015). Exploring intensive longitudinal measures of student engagement in blended learning. Int. Re. Res. Open. Distri. Learn. 16, 131–155. doi: 10.19173/irrodl.v16i3.2015

[ref52] Hill F. M. (1995). Managing service quality in higher education: the role of the student as primary consumer. Qual. Assur. Educ. 3, 10–21. doi: 10.1108/09684889510093497

[ref53] Hmelo-Silver C. E. (2004). Problem-based learning: what and how do students learn? Educ. Psychol. Rev. 16, 235–266. doi: 10.1023/B:EDPR.0000034022.16470.f3

[ref54] Hmelo-Silver C. E. (2006). Barrows HS. Goals and strategies of a problem-based learning facilitator. Interdisc. J. PBL. Learn. 1, 21–39. doi: 10.7771/1541-5015.1004

[ref55] Ho V. T. Nakamori Y. Ho T. B. Lim C. P. (2016). Blended learning model on hands-on approach for in-service secondary school teachers: combination of E-learning and face-to-face discussion. Educ. Inf. Technol. 21, 185–208. doi: 10.1007/s10639-014-9315-y

[ref56] Hua M. Wang L. (2023). The relationship between Chinese university students’ learning preparation and learning achievement within the EFL blended teaching context in COVID-19 post-epidemic era: the mediating effect of learning methods. PLoS One 18:e0280919. doi: 10.1371/journal.pone.0280919, PMID: 36693072 PMC10045568

[ref57] Hwang E. H. Kim K. H. (2023). Relationship between optimism, emotional intelligence, and academic resilience of nursing students: the mediating effect of self-directed learning competency. Front. Public Health 11:1182689. doi: 10.3389/fpubh.2023.1182689, PMID: 37275498 PMC10234118

[ref58] Iverson R. D. Maguire C. (2000). The relationship between job and life satisfaction: evidence from a remote mining community. Hum. Relat. 53, 807–839. doi: 10.1177/0018726700536003

[ref59] Jiang Y. Chen Y. Lu J. Wang Y. (2021). The effect of the online and offline blended teaching mode on English as a foreign language learners’ listening performance in a Chinese context. Front. Psychol. 12:742742. doi: 10.3389/fpsyg.2021.742742, PMID: 34867623 PMC8634958

[ref60] Jiang P. Wijaya T. T. Mailizar M. Zulfah Z. Astuti A. (2022). How micro-lectures improve learning satisfaction and achievement: a combination of ECM and extension of TAM models. Mathematics 10:3430. doi: 10.3390/math10193430

[ref61] Kanashiro P. Iizuka E. S. Sousa C. Dias S. E. F. (2020). Sustainability in management education: a Biggs’ 3P model application. Educ. Int. J. Sustain. High. Educ. 21, 671–684. doi: 10.1108/IJSHE-05-2019-0176

[ref62] Kaplan A. M. Haenlein M. (2016). Higher education and the digital revolution: about MOOCs, SPOCs, social media, and the cookie monster. Bus. Horiz. 59, 441–450. doi: 10.1016/j.bushor.2016.03.008

[ref63] Keengwe J. Kang J. J. (2013). A review of empirical research on blended learning in teacher education programs. Educ. Inf. Technol. 18, 479–493. doi: 10.1007/s10639-011-9182-8

[ref64] Kim D. Lee M. (2019). The structural relationship among smartphone dependency, teaching presence, deep approach to learning and satisfaction in online deeper learning, in Proceedings of the 8th International Conference on Educational and Information Technology. New York: ACM Digital Library, 27–32.

[ref65] King S. E. Cerrone K. A. (2012). Blended learning environments in higher education: a case study of how professors make it happen. Mid-W. Educ. Res. 25, 44–59.

[ref66] Kintu M. J. Zhu C. Kagambe E. (2017). Blended learning effectiveness: the relationship between student characteristics, design features and outcomes. Int. J. Educ. Technol. High. Educ. 14, 1–20. doi: 10.1186/s41239-017-0043-4

[ref67] Korr J. Derwin E. B. Greene K. Sokoloff W. (2012). Transitioning an adult-serving university to a blended learning model. J. Contin. High. Educ. 60, 2–11. doi: 10.1080/07377363.2012.649123

[ref68] Köse U. (2010). A blended learning model supported with web 2.0 technologies. Proc. Soc. Behav. Sci. 2, 2794–2802. doi: 10.1016/j.sbspro.2010.03.417

[ref69] Law K. M. Lee V. C. Yu Y. T. (2010). Learning motivation in e-learning facilitated computer programming courses. Comput. Educ. 55, 218–228. doi: 10.1016/j.compedu.2010.01.007

[ref70] Lee K. Tsai P. S. Chai C. S. Koh J. H. L. (2014). Students’ perceptions of self-directed leaning and collaborative learning with and without technology. J. Comput. Assist. Learn. 30, 425–437. doi: 10.1111/jcal.12055

[ref71] Li H. Liu J. Q. (2023). Research on general education curriculum construction in Normal universities from perspective of high-quality development. Heilongjiang Res. Higher Edu. 4:5. doi: 10.19903/j.cnki.cn23-1074/g.2023.04.005

[ref72] Linkous H. M. (2021). Self-directed learning and self-regulated learning: what’s the difference? A literature analysis. Am. Assoc. Adult Cont. Educ. 10, 27–30.

[ref73] Liu H. Yao M. L. Li J. Li R. (2021). Multiple mediators in the relationship between perceived teacher autonomy support and student engagement in math and literacy learning. Educ. Psychol. 41, 116–136. doi: 10.1080/01443410.2020.1837346

[ref74] Llorente A. M. P. Gómez M. C. S. García-Peñalvo F. J. (2016). Assessing the effectiveness of interactive and collaborative resources to improve reading and writing in English. Int. J. Hum. Cap. Inf. Technol. Prof. 7, 66–85. doi: 10.4018/IJHCITP.2016010105

[ref75] Long H. B. (1985). Contradictory expectations? Achievement and satisfaction in adult learning. The J. Contin. High. Educ. 33, 10–12. doi: 10.1080/07377366.1985.10401035

[ref76] López-Pérez M. V. Pérez-López M. C. Rodríguez-Ariza L. (2011). Blended learning in higher education: students’ perceptions and their relation to outcomes. Comput. Educ. 56, 818–826. doi: 10.1016/j.compedu.2010.10.023

[ref77] Lucas U. (2001). Deep and surface approaches to learning within introductory accounting: a phenomenographic study. Account. Educ. 10, 161–184. doi: 10.1080/09639280110073443

[ref78] Ma L. Luo H. Xiao L. (2021). Perceived teacher support, self-concept, enjoyment and achievement in reading: a multilevel mediation model based on PISA 2018. Learn. Individ. Differ. 85:101947. doi: 10.1016/j.lindif.2020.101947

[ref79] MacKinnon D. P. Lockwood C. M. Williams J. (2004). Confidence limits for the indirect effect: distribution of the product and resampling methods. Multivar. Behav. Res. 39, 99–128. doi: 10.1080/07294360903146841, PMID: 20157642 PMC2821115

[ref80] Mahaye N. E. (2020). The impact of COVID-19 pandemic on education: navigating forward the pedagogy of blended learning. Res. Online 5, 4–9.

[ref81] Mali D. Lim H. (2021). How do students perceive face-to-face/blended learning as a result of the Covid-19 pandemic? Int. J. Manag. Educ. 19:100552. doi: 10.1016/j.ijme.2021.100552

[ref82] Marsh H. Trautwein U. Lüdtke O. (2005). Academic self-concept, interest, grades, and standardized test scores: reciprocal effects models of causal ordering. Child Dev. 76, 397–416. doi: 10.1111/j.1467-8624.2005.00853.x, PMID: 15784090

[ref83] Milligan C. Littlejohn A. (2014). Supporting professional learning in a massive open online course. Int. Rev. Res. Open Dist. Learn. 15, 197–213. doi: 10.19173/irrodl.v15i5.1855

[ref84] Miyazoe T. Anderson T. (2010). Learning outcomes and students’ perceptions of online writing: simultaneous implementation of a forum, blog, and wiki in an EFL blended learning setting. System 38, 185–199. doi: 10.1016/j.system.2010.03.006

[ref85] Mladenovic R. (2000). An investigation into ways of challenging introductory accounting students’ negative perceptions of accounting. Account. Educ. 9, 135–155. doi: 10.1080/09639280010000147

[ref86] Mossholder K. W. Bennett N. Kemery E. R. (1998). Relationships between bases of power and work reactions: the mediational role of procedural justice. J. Manag. 24, 533–552. doi: 10.1016/S0149-2063(99)80072-5

[ref87] Murphy L. Eduljee N. B. Croteau K. Parkman S. (2020). Relationship between personality type and preferred teaching methods for undergraduate college students. Int. J. Res. Educ. Sci. 6, 100–109. doi: 10.46328/ijres.v6i1.690

[ref88] Nakamura J. Csikszentmihalyi M. (2009). “Flow theory and research” in The Oxford handbook of positive psychology. ed. Shane J. L. (New York, NY: Oxford University Press), 195–206.

[ref89] Osgerby J. (2013). Students’ perceptions of the introduction of a blended learning environment: an exploratory case study. Account. Educ. 22, 85–99. doi: 10.1080/09639284.2012.729341

[ref90] Podsakoff N. P. (2003). Common method biases in behavioral research: a critical review of the literature and recommended remedies. J. Appl. Psychol. 88, 879–903. doi: 10.1037/0021-9010.88.5.879, PMID: 14516251

[ref91] Podsakoff P. M. Organ D. W. (1986). Self-reports in organizational research: problems and prospects. J. Manag. 12, 531–544. doi: 10.1177/014920638601200408

[ref92] Reid A. Wood L. N. Smith G. H. Petocz P. (2013). Intention, approach and outcome: university mathematics students’ conceptions of learning mathematics. Int. J. Sci. Math. Educ. 3, 567–586. doi: 10.1007/s10763-004-5818-0

[ref93] Rigdon E. E. Sarstedt M. Ringle C. M. (2017). On comparing results from CB-SEM and PLS-SEM: five perspectives and five recommendations. Marketing 39, 4–16. doi: 10.15358/0344-1369-2017-3-4

[ref94] Sáiz-Manzanares M. C. Escolar-Llamazares M. C. Arnaiz González Á. (2020). Effectiveness of blended learning in nursing education. Int. J. Envir. Res. Public Health 17:1589. doi: 10.3390/ijerph17051589, PMID: 32121514 PMC7084479

[ref95] Scardamalia M. Bereiter C. McLean R. S. Swallow J. Woodruff E. (1989). Computer-supported intentional learning environments. J. Educ. Comput. Res. 5, 51–68. doi: 10.2190/CYXD-6XG4-UFN5-YFB0

[ref96] Schunk D. H. DiBenedetto M. K. (2020). Motivation and social cognitive theory. Contemp. Educ. Psychol. 60:101832. doi: 10.1016/j.cedpsych.2019.101832

[ref01] Shen P. D. Lee T. H. Tsai C. W. (2011). Applying blended learning with web-mediated self-regulated learning to enhance vocational Students’ computing skills and attention to learn. Interact. learn. Envir. 19, 193–209. doi: 10.1080/10494820902808958

[ref97] Shen D. Cho M. H. Tsai C. L. Marra R. (2013). Unpacking online learning experiences: online learning self-efficacy and learning satisfaction. Internet High. Educ. 19, 10–17. doi: 10.1016/j.iheduc.2013.04.001

[ref98] Shrout P. E. Bolger N. (2002). Mediation in experimental and non-experimental studies: new procedures and recommendations. Psychol. Methods 7, 422–445. doi: 10.1037/1082-989X.7.4.422, PMID: 12530702

[ref99] Sitthiworachart J. Joy M. Mason J. (2021). Blended learning activities in an e-business course. Educ. Sci. 11:763. doi: 10.3390/educsci11120763

[ref100] Smith P. (2015). Blended learning: it’ s not the tech, it’s how the tech is used Available at: https://www.rocketshipschools.org/blended-learning-its-not-the-tech-its-how-the-tech-is-used/ (Accessed February 19, 2015).

[ref101] So H. J. Brush T. A. (2008). Student perceptions of collaborative learning, social presence and satisfaction in a blended learning environment: relationships and critical factors. Comput. Educ. 51, 318–336. doi: 10.1016/j.compedu.2007.05.009

[ref102] Sun P. C. Tsai R. J. Finger G. Chen Y. Y. Yeh D. (2008). What drives a successful e-learning? An empirical investigation of the critical factors influencing learner satisfaction. Comput. Educ. 50, 1183–1202. doi: 10.1016/j.compedu.2006.11.007

[ref103] Syahrawati E. Y. Susantini E. Indana S. (2022). Profile of blended learning implementation in learning activities. IJORER: Int. J. Recent Educ. Res. 3, 45–60. doi: 10.46245/ijorer.v3i1.183

[ref104] Tadesse S. G. Tadesse D. G. Dagnaw E. H. (2022). Problem based learning approach increases the learning satisfaction of health science students in Ethiopian universities: a comparative cross sectional study. BMC Med. Educ. 22:334. doi: 10.1186/s12909-022-03397-5, PMID: 35501812 PMC9063231

[ref105] Trigwell K. Ashwin P. Millan E. S. (2013). Evoked prior learning experience and approach to learning as predictors of academic achievement. Br. J. Educ. Psychol. 83, 363–378. doi: 10.1111/j.2044-8279.2012.02066.x 23822526

[ref106] Utts J. Sommer B. Acredolo C. Maher M. W. Matthews H. R. (2017). A study comparing traditional and hybrid internet-based instruction in introductory statistics classes. J. Stat. Educ. 11, 1–14. doi: 10.1080/10691898.2003.11910722

[ref107] Vaughan N. D. Cleveland-Innes M. Garrison D. R. (2013). Teaching in blended learning environments: creating and sustaining communities of inquiry. Edmonton: AU Press.

[ref108] Vermunt J. D. Donche V. (2017). A learning patterns perspective on student learning in higher education: state of the art and moving forward. Educ. Psychol. Rev. 29, 269–299. doi: 10.1007/s10648-017-9414-6

[ref109] Wan K. Zheng X. Ren Y. (2020). Is online learning at scale ready? After the outbreak period of online learning and intelligent technology application (in Chinese). J. Dist. Educ. 38, 105–112. doi: 10.15881/j.cnki.cn33-1304/g4.2020.03.011

[ref110] Wijaya T. T. Cao Y. Bernard M. Rahmadi I. F. Lavicza Z. Surjono H. D. (2022). Factors influencing microgame adoption among secondary school mathematics teachers supported by structural equation modelling-based research. Front. Psychol. 13:952549. doi: 10.3389/fpsyg.2022.952549, PMID: 36160545 PMC9493482

[ref111] Williamson S. N. (2007). Development of a self-rating scale of self-directed learning. Nurse Res. 14, 66–83. doi: 10.7748/nr2007.01.14.2.66.c6022, PMID: 17315780

[ref112] Woltering V. Herrler A. Spitzer K. Spreckelsen C. (2009). Blended learning positively affects students’ satisfaction and the role of the tutor in the problem-based learning process: results of a mixed-method evaluation. Adv. Health Sci. Tduc. Theory Pract. 14, 725–738. doi: 10.1007/s10459-009-9154-6, PMID: 19184497

[ref113] Xiao J. (2016). Who am I as a distance tutor? An investigation of distance tutors’ professional identity in China. Dist. Educ. 37, 4–21. doi: 10.1080/01587919.2016.1158772

[ref114] Yang Y. F. Kuo N. C. (2021). Blended learning to foster EFL college students’ global literacy. Comput. Assist. Lang. Learn. 36, 81–102. doi: 10.1080/09588221.2021.1900874

[ref115] Yin H. Lu G. Wang W. (2014). Unmasking the teaching quality of higher education: students’ course experience and approaches to learning in China. Assess. Eval. High. Educ. 39, 949–970. doi: 10.1080/02602938.2014.880107

[ref116] Yin H. Wang W. Han J. (2016). Chinese undergraduates’ perceptions of teaching quality and the effects on approaches to studying and course satisfaction. High. Educ. 71, 39–57. doi: 10.1007/s10734-015-9887-5

[ref117] Zhang M. L. (2017). The impact of English self-directed learning ability on the satisfaction of blended cooperative learning (in Chinese). Mod. Foreign Lang 40, 564–574 +585.

[ref118] Zhang S. Ma R. Wang Z. Li G. And Fa T. (2022). Academic self-concept mediates the effect of online learning engagement on deep learning in online courses for Chinese nursing students: a cross-sectional study. Nurse Educ. Today:105481. doi: 10.1016/j.nedt.2022.105481 35872403

[ref119] Zhao T. (2022). Research on the construction and practice of blended learning model supported by intelligent technology (in Chinese). China Educ. Technol. 9, 137–142.

[ref120] Zhao W. Song Y. Zhao Q. (2019). The effect of teacher support on primary school students’ reading engagement: the mediating role of reading interest and Chinese academic self-concept. Educ. Psychol. 39, 236–253. doi: 10.1080/01443410.2018.1497146

[ref121] Zhao J. Wijaya T. T. Mailizar M. Habibi A. (2022). Factors influencing student satisfaction toward STEM education: exploratory study using structural equation modeling. Appl. Sci. 12:9717. doi: 10.3390/app12199717

[ref122] Zhao G. D. Yuan S. (2010). A study on student satisfaction and its influencing factors in blended learning: a case study of Peking University teaching network (in Chinese). China Dist. Educ. 370, 32–38 +79. doi: 10.13541/j.cnki.chinade.2010.06.003

