# Supplementary material for: The impact of self-directed learning experience and course experience on learning satisfaction of university students in blended learning environments: the mediating role of deep and surface learning approach
Source: Front Psychol. 2024 Jan 8;14:1278827. doi: 10.3389/fpsyg.2023.1278827 (PMC10801907; doi:10.3389/fpsyg.2023.1278827)
Supplement: Supplementary file 2 [file Presentation_1.pdf]

Appendix 1

Self-Rating Scale of Blended Self-Directed Learning(SRSBSDL)

Response Key: 1 = Never; 2 =Rarely; 3 = Sometimes; 4 = Often; 5 = Very often; 6 =Always

1	**Awareness**	1	2	3	4	5	6
1.1	I am able to select the best method for my own blended learning.						
1.2	I consider teachers as facilitators of learning rather than providing information only in blended learning.						
1.3	I keep up to date on different resources available in blended learning.						
1.4	I am responsible for identifying my areas of deficit in blended learning.						
1.5	I relate my experience with new information in blended learning.						
1.6	I am able to maintain self-motivation in blended learning.						
2	**Learning Strategies**	1	2	3	4	5	6
2.1	I participate in group discussions and I find peer coaching effective in blended learning.						
2.2	I find interactive teaching-learning sessions effective in blended learning.						
2.3	I regard problems as challenges in blended learning.						
2.4	I arrange my blended self-learning routine in such a way that it helps develop a permanent learning culture in my life.						
2.5	I find modern educational technology enhances my blended learning process.						
2.6	I am able to decide my own learning strategy in blended learning.						
3	**learning activity**	1	2	3	4	5	6
3.1	I raise relevant question(s) in blended teaching-learning sessions.						
3.2	I analyse and critically reflect on new ideas, information or any learning experiences in blended learning.						
3.3	I rehearse and revise new lessons in blended learning.						
3.4	My concentration intensifies and I become more attentive when I read a complex study content in blended learning.						
3.5	I identify the important points when reading a chapter or an article in blended learning.						
3.6	I keep annotated notes or a summary of my many ideas, reflections and new learning in blended learning.						
4	**Evaluation**	1	2	3	4	5	6
4.1	I self-assess before I get feed back from instructors in blended learning.						
4.2	I identify the areas for further development in whatever I have accomplished in blended learning.						
4.3	I am able to monitor my learning progress and whether I have accomplished my goals in blended learning.						
4.4	I find both success and failure inspire me to further move on in blended learning.						
4.5	I review and reflect on my blended learning activities.						
4.6	I appreciate when my work can be peer reviewed in blended learning.						
5	**Interpersonal skills**	1	2	3	4	5	6
5.1	I maintain good interpersonal relationships with others in blended learning.						
5.2	I am able to identify my role and to to work collaboratively within a group in blended learning.						

5.3	My interaction with others helps me to develop the insight to plan for further blended learning.						
5.4	I need to share information with others in blended learning.						
5.5	I make use of any opportunities I come across in blended learning.						
5.6	I am able to express my views freely in blended learning.						

Blended Course Experience Questionnaire (BCEQ)

Response Key: 1 = Strongly disagree；2 =Disagree; 3 = Neutral; 4 = Agree; 5 = Strongly agree

1	**Good teaching(GT)**	1	2	3	4	5
1.1	The teaching staff of the blended courses motivated me to do my best work when I am in blended learning.					
1.2	The teaching staff who used blended teaching put a lot of time into commenting on my work.					
1.3	The staff who used blended teaching made a real effort to understand difficulties I might be having in blended learning.					
1.4	The teaching staff normally gave me helpful feedback on how I was going when I am in blended learning.					
1.5	My lecturers were extremely good at explaining things in blended classes.					
1.6	The teaching staff who used blended teaching worked hard to make their subjects interesting.					
2	**General Skills(GS)**	1	2	3	4	5
2.1	The blended courses developed my problem solving skills.					
2.2	The blended courses sharpened my analytic skills.					
2.3	The blended courses helped me develop my ability to work as a team member.					
2.4	As a result of the blended courses, I feel confident about tackling unfamiliar problems.					
2.5	The blended courses improved my skills in written communication.					
2.6	The blended courses helped me to develop the ability to plan my own work.					
3	**Clear Goals and Standard(CGS)**	1	2	3	4	5
3.1	It was always easy to know the standard of work expected in blended courses.					
3.2	It was often easy to discover what was expected of me in blended courses.					
3.3	I usually had a clear idea of where I was going in blended courses.					
3.4	The teaching staff who used blended teaching made it clear right from the start what they expected from students.					
4	**Overall Course Experience(Overall-CE)**	1	2	3	4	5
4.1	Overall, I was satisfied with the quality of the blended courses.					

Quesionnaire of Deep Approach to Blended Learning(QDA-BL)

Response Key: 1 = Never; 2 =Hardly ever; 3 =Rarely; 4 = Sometimes; 5 = Frequently; 6 = Very frequently; 7 = Always

	**QDA-BL**	1	2	3	4	5	6	7
1	I feel that virtually any topic can be highly interesting once I get into it in blended learning environment.							
2	I come to blended classes with questions in mind that I want answering.							
3	I find that I have to do enough work on a topic so that I can form my own							

	conclusions before I am satisfied in blended learning.							

Questionnaire of Surface Approach to Blended Learning(QSA-BL)

Response Key: 1 = Never; 2 =Rarely; 3 = Sometimes; 4 = Often; 5 =Always

	**QSA-BL**	1	2	3	4	5
1	My aim is to pass blended courses while doing as little work as possible.					
2	I do not find my blended courses very interesting so I keep my work to the minimum.					
3	I generally restrict my study to what is specifically set as I think it is unnecessary to do anything extra in blended learning.					
